# GluN2D-containing NMDA receptors enhance temporal integration in VIP neurons in the inferior colliculus

**DOI:** 10.1101/2023.04.27.538607

**Published:** 2023-04-28

**Authors:** Audrey C. Drotos, Yoani N. Herrera, Rachel L. Zarb, Michael T. Roberts

**Affiliations:** 1. Kresge Hearing Research Institute, Department of Otolaryngology – Head and Neck Surgery, University of Michigan, Ann Arbor, Michigan 48109; 2. Department of Molecular and Integrative Physiology, University of Michigan, Ann Arbor, Michigan 48109

## Abstract

Along the ascending auditory pathway, there is a broad shift from temporal coding, which is common in the lower auditory brainstem, to rate coding, which predominates in auditory cortex. This temporal-to-rate transition is particularly prominent in the inferior colliculus (IC), the midbrain hub of the auditory system, but the mechanisms that govern how individual IC neurons integrate information across time remain largely unknown. Here, we report the widespread expression of *GluN2C* and *GluN2D* mRNA in IC neurons. GluN2C/D-containing NMDA receptors are relatively insensitive to voltage-dependent Mg^2+^ block, and thus can activate at resting membrane potential. Using in situ hybridization and pharmacology, we show that VIP neurons in the IC express GluN2D-containing NMDA receptors that are activatable by ascending input from T-stellate cells in the anteroventral cochlear nucleus and commissural inputs from the contralateral IC. In addition, GluN2D-containing receptors have much slower kinetics than other NMDA receptors, and we found that GluN2D-containing receptors facilitate temporal summation in VIP neurons by prolonging the time window for synaptic integration. These results suggest that GluN2C/D-containing NMDA receptors support the shift from temporal to rate coding in the auditory system by facilitating the integration of ascending inputs.

## Introduction

The inferior colliculus (IC) in the midbrain plays a critical role in auditory processing, functioning as a hub of integration in the auditory pathway (Adams, 1979; Oliver and Huerta, 1992) and as a site where phase-locked ascending inputs are transformed into a firing rate code (Hewitt and Meddis, 1994; Joris et al., 2004). However, the cellular and circuit mechanisms that govern how IC neurons integrate synaptic inputs and convert temporal to rate codes remain largely unknown. In other brain regions, the slow kinetics of NMDA receptors (NMDARs) can play a critical role in temporal integration of synaptic inputs (D’Angelo et al., 1995; Lo et al., 2013). Intriguingly, previous studies have found that many IC neurons express NMDARs that are activated at resting membrane potential. For example, in brain slice recordings from 9–16-day-old rats, stimulation of the lateral lemniscus resulted in NMDAR activation even in the presence of AMPA receptor (AMPAR) antagonists (Kelly and Zhang, 2002; Ma et al., 2002; Wu, 2004). Similarly, we recently found in adult mice that optogenetic stimulation of commissural inputs, but not inputs from the dorsal cochlear nucleus (DCN), elicited excitatory postsynaptic potentials (EPSPs) in IC VIP neurons that had a significant contribution from NMDARs at resting membrane potential (Goyer et al., 2019).

These results suggest that NMDARs that conduct current at resting membrane potential are a common feature of IC circuits, but this is unusual. At most glutamatergic synapses, activation of AMPARs is required to depolarize the membrane potential and remove voltage-dependent Mg^2+^ block of NMDARs, allowing NMDARs to contribute to the response. One potential explanation for this phenomenon is that NMDARs in IC neurons may be less sensitive to Mg^2+^ block due to their subunit composition (Paoletti et al., 2013). NMDARs are heterotetramers comprised of two obligatory GluN1 subunits and a combination of GluN2(A-D) and/or GluN3(A-B) subunits (Traynelis et al., 2010). A residue in the GluN2 subunit confers Mg^2+^ sensitivity (Monyer et al., 1992; Siegler Retchless et al., 2012), with GluN2C/D subunit-containing receptors being much less sensitive to Mg^2+^ block than GluN2A/B subunit-containing receptors. Inclusion of GluN2C/D subunits in NMDARs thus gives rise to receptors with significant currents at resting membrane potential, as has been demonstrated in the mouse barrel cortex (Binshtok et al., 2006).

In addition to their differences in Mg^2+^ sensitivity, NMDARs containing GluN2C and GluN2D subunits have slower kinetics than NMDARs with GluN2A and GluN2B subunits (Paoletti et al., 2013). These slow kinetics can expand the time window for temporal summation of inputs, as seen in intracortical inputs to cortical pyramidal cells, where GluN2C/D-containing receptors integrate inputs more efficiently than GluN2A/B-containing receptors (Kumar and Huguenard, 2003).

Here, we tested whether GluN2C/D-containing NMDARs mediate NMDAR currents in the IC and the role of these receptors in synaptic integration. We found that IC VIP neurons, a class of excitatory principal neurons (Goyer et al., 2019), express NMDARs that activate at resting potential. We show that VIP neurons express GluN2D mRNA and that many other IC neurons express GluN2C and/or GluN2D mRNA, pointing to a prominent role for GluN2C/D-containing NMDARs in the IC. NMDAR-mediated responses in VIP neurons were sensitive to GluN2C/D-specific pharmacology, suggesting that NMDAR activation at resting potential in IC neurons is due to activation of receptors containing GluN2C/D subunits. Using channelrhodopsin-assisted circuit mapping, we demonstrate that GluN2C/D-containing NMDARs in VIP neurons are activated by both ascending inputs from the anteroventral cochlear nucleus (AVCN) and by inputs from the contralateral IC. Additionally, GluN2C/D-specific antagonists decreased temporal summation of commissural inputs in VIP neurons, indicating that GluN2C/D-containing NMDARs promote synaptic integration in IC neurons. Overall, this work provides the first evidence of a cellular mechanism for NMDAR activation at resting potential in IC neurons and shows that the widespread expression of GluN2C/D subunits in the IC could play an important role in the transition from temporal to rate codes in the IC.

## Results

### Ascending inputs from the anteroventral cochlear nucleus activate NMDARs in IC VIP neurons at resting potential

**Figure 1.**
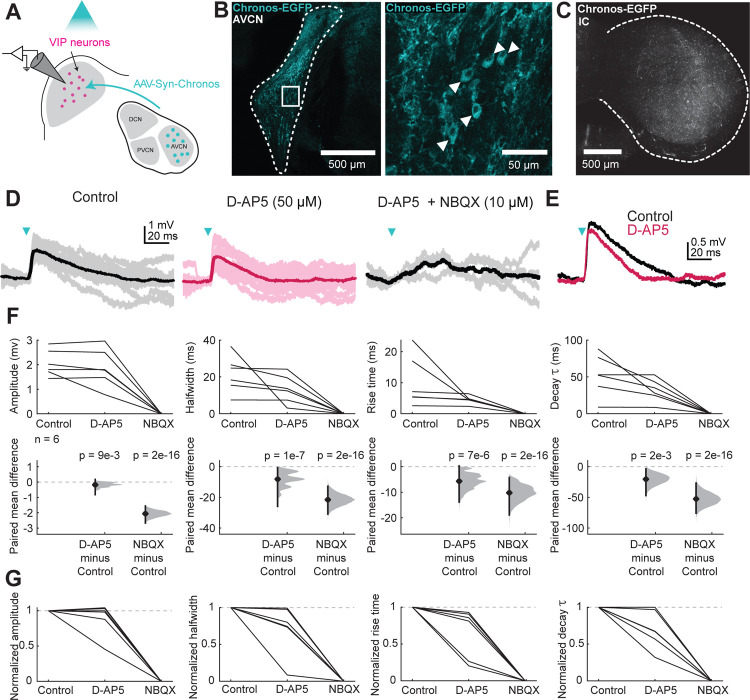
T-stellate projections to VIP neurons activate NMDARs at resting potential **A**, Viral injections in the AVCN drove Chronos expression in T-stellate cell projections to the IC. Optogenetic circuit mapping and pharmacology were used to examine postsynaptic responses in VIP neurons. **B**, Confocal images showing Chronos-eGFP expression in the right AVCN. **C**, eGFP-labeled axons and terminals formed a wide band of expression in the left IC. **D**, Brief (2.5 ms) flashes of blue light (blue arrow) elicited EPSPs in a VIP neuron (left); the halfwidth of this EPSP was reduced by application of D-AP5 (middle) and completely abolished by application of both D-AP5 and NBQX (right). **E**, Application of D-AP5 caused a decrease in the halfwidth of the representative cell from D. **F**, D-AP5 decreased the amplitude (LMM: β = −0.25, 95% CI [−0.44, −0.064], *p* = 0.0093, n = 6), halfwidth (β = −6.49, 95% CI [−8.84, −4.15], *p* = 1.36e-07, n = 6), rise time (β = −3.87, 95% CI [−5.52, −2.21], *p* = 7.36e-06, n = 6), and decay τ (β = −12.84, 95% CI [−20.76, −4.83], *p* = 0.0018 n = 6) of the response to optogenetic activation of T-stellate inputs. NBQX completely abolished the response in all cases (amplitude (β = −2.09, 95% CI [−2.27, −1.91], *p* = 2e-16, n = 6), halfwidth (β = −19.64, 95% CI [−21.87, −17.41], *p* = 2e-16, n = 6), rise time (β = −8.73, 95% CI [−10.28, −7.14], *p* = 2e-16, n = 6), and decay τ (β = −43.79, 95% CI [−50.99, −36.53], *p* = 2e-16, n = 6). Vertical lines on the paired mean difference plots indicate 95% bootstrap confidence intervals. **G**, Normalized amplitude, halfwidth, rise time, and decay time constant plots from F.

T-stellate neurons in the anteroventral cochlear nucleus (AVCN) are one of only three neuron types in the brain that receive synaptic input from the auditory nerve and directly project to the IC (Oertel et al., 2011). Despite the likely importance of such a privileged information route for auditory processing, how T-stellate synapses influence the excitability of IC neurons has remained unknown. The difficulty has been that axons from numerous sources intermingle in the IC, rendering electrical stimulation highly non-specific. However, we recently showed that this problem can be overcome using targeted viral transfections and optogenetic circuit mapping in brain slices (Goyer et al., 2019; Goyer and Roberts, 2020). While using this approach to examine T-stellate input to VIP neurons in the IC, we found that T-stellate synapses elicited EPSPs that often had a significant NMDAR component at or near resting membrane potential (Figure 1).

For these experiments, we injected AAV5.Syn.Chronos-GFP.WPRE.bGH into the right AVCN of VIP-IRES-Cre × Ai14 mice to drive expression of the excitatory opsin Chronos in T-stellate projections to the IC (Figure 1A–C). Since T-stellate cells are the only AVCN neurons that project to the IC, this allowed us to use light to selectively stimulate T-stellate synapses in IC brain slices. We then performed whole-cell patch-clamp recordings targeted to fluorescently labeled VIP neurons in the IC (Figure 1A). We found that 2 – 5 ms flashes of blue light elicited EPSPs in VIP neurons (Figure 1D). Application of the NMDAR antagonist D-AP5 significantly reduced the amplitude (LMM, β = −0.25, *p* = 0.0093), halfwidth (β = −6.49, p = 1.36e-07), 10–90% rise time (β = −3.87, *p* = 7.36e-06), and time constant (β = −12.84, *p* = 0.0018) of the elicited EPSP (Figure 1E, F), consistent with the hypothesis that NMDARs on these neurons contribute to responses at resting potentials. These data, together with our previously published work (Goyer et al., 2019), show that ascending synapses from the AVCN and commissural synapses from the contralateral IC elicit NMDAR-mediated responses in IC VIP neurons at resting membrane potential.

### NMDAR currents in VIP neurons are sensitive to GluN2C/D-specific drugs

NMDAR currents at resting potential are unusual, as most NMDARs in the adult brain have a voltage-dependent Mg^2+^ block which prevents ions from flowing through the channel pore at or near resting potential (Traynelis et al., 2010; Paoletti et al., 2013). However, sensitivity to Mg^2+^ block depends on the subunit composition of the receptor. While receptors containing GluN2A/B subunits are highly sensitive to Mg^2+^ block, receptors containing GluN2C/D subunits are less sensitive, allowing them to conduct current even at hyperpolarized potentials (Siegler Retchless et al., 2012). Based on this, we hypothesized that VIP neurons express NMDARs containing GluN2C and/or GluN2D subunits.

**Figure 2.**
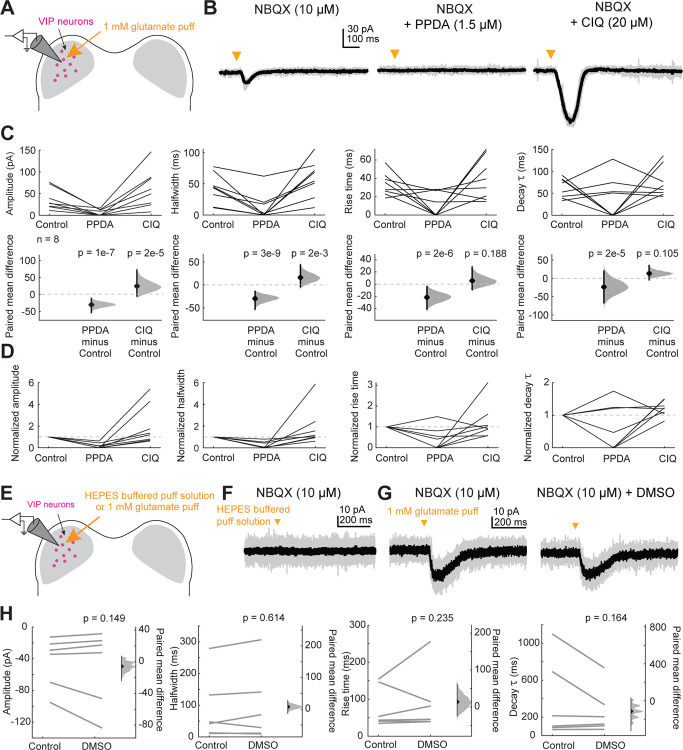
VIP neurons express GluN2C/D-containing NMDARs **A**, Puffs of glutamate onto VIP neurons activated NMDARs. Pharmacology was used to examine whether GluN2C/D-containing receptors contributed to elicited currents. **B**, Brief (10 ms) puffs of glutamate onto a VIP neuron elicited a small inward current (left) which was completely abolished by the GluN2C/D antagonist PPDA (middle) and enhanced by the GluN2C/D positive allosteric modulator CIQ (right). **C**, PPDA decreased the amplitude (LMM: β = −29.95, 95% CI [−40.35, −19.57], *p* = 1.44e-07, n = 8), halfwidth (β = −33.79, 95% CI [−43.92, −23.58], *p* = 2.85e-09, n = 8), rise time (β = −23.71, 95% CI [−32.80, −14.51], *p* = 1.65e-06, n = 8), and decay τ (β = −36.78, 95% CI [−52.91, −20.74], *p* = 2.08e-05, n = 8) of the response to the glutamate puff. CIQ enhanced the amplitude (β = 24.13, 95% CI [13.65, 34.59], *p* = 1.69e-05, n = 8) and halfwidth (β = 16.88, 95% CI [6.68, 27.18], *p* = 0.0017, n = 8) but did not alter the rise time (β = 6.19, 95% CI [−2.90, 15.40], *p* = 0.19, n = 8) or decay τ (β = 13.31, 95% CI [−2.67, 29.22], *p* = 0.11, n = 8) of the response to the glutamate puff. **D**, Normalized amplitude, halfwidth, rise time, and decay time constant plots from C. **E**, Control puffs of vehicle solution and control DMSO wash-ins to examine whether the vehicle solution and/or DMSO caused a response in the cell or influenced the EPSP. **F**, A brief (10 ms) puff of the HEPES-buffered puff solution did not elicit a response in any of the VIP neurons tested (n = 6). **G**, A VIP neuron response to a brief (10 ms) puff of glutamate (left) was unchanged after bath application of DMSO (right, vehicle for PPDA and CIQ). **H**, Application of DMSO did not alter EPSC amplitude (LMM: β = −5.57, 95% CI [−13.14, 1.95], *p* = 0.15, n = 6), halfwidth (β = 5.41, 95% CI [−15.60, 26.57], *p* = 0.61, n = 6), rise time (β = 13.45, 95% CI [−8.67, 35.62], *p* = 0.23, n = 6), or decay τ (β = −81.17, 95% CI [−195.07, 32.15], *p* = 0.16, n = 6).

To test this hypothesis, we voltage-clamped VIP neurons at −70 mV and used a puffer pipette containing 1 mM glutamate to elicit EPSCs (Figure 2A). We found that 10 ms glutamate puffs reliably elicited EPSCs in VIP neurons (Figure 2B). We next performed puffs in the presence of 1.5 μM PPDA, a GluN2C/D-selective antagonist (Lozovaya et al., 2004; Li et al., 2020; Jing et al., 2022), and then in the presence of 20 μM CIQ, a GluN2C/D-selective positive allosteric modulator (Feng et al., 2014; Zhang et al., 2014; Nouhi et al., 2018; Liu et al., 2021). PPDA significantly decreased the amplitude (LMM: β = −29.95, *p* = 1.44e-07), halfwidth (β = −33.79, p = 2.85e-09), rise time (β = −23.71, *p* = 1.64e-06) and decay time constant (β = −36.78, *p* = 2.08e-05) of the elicited EPSCs, while CIQ significantly enhanced the amplitude (β = 24.13, *p* = 1.69e-05) and halfwidth (β = 16.88, *p* = 0.0017) (Figure 2C,D). These results suggest that at least a portion of the NMDARs expressed in VIP neurons contain GluN2C/D subunits.

### VIP neurons in adult mice express *GluN2D* but rarely *GluN2C* mRNA

GluN2C and GluN2D subunits are similar in their sensitivity to Mg^2+^ block, but expression levels of the *GluN2C* and *GluN2D* mRNA changes dramatically during development (Paoletti et al., 2013). GluN2D-containing NMDARs (along with GluN2B-containing NMDARs) are primarily found early in development (Wenzel et al., 1996) and are largely replaced with GluN2A and GluN2C subunits by adulthood (Paoletti et al., 2013), with some exceptions in small populations of interneurons, such as those found in hippocampus (Monyer et al., 1992). In many cases, the developmental switch in NMDAR subunit composition is thought to be driven by sensory experience (Traynelis et al., 2010). Expression of GluN2 subunits is also spatially regulated: while GluN2A and GluN2B-containing NMDARs are typically found at the synapse, GluN2C and GluN2D-containing NMDARs are often found at extrasynaptic sites (Paoletti et al., 2013), although there exists growing evidence for their involvement in synaptic transmission, such as in striatum (Logan et al., 2007) and substantia nigra (Brothwell et al., 2008).

**Figure 3.**
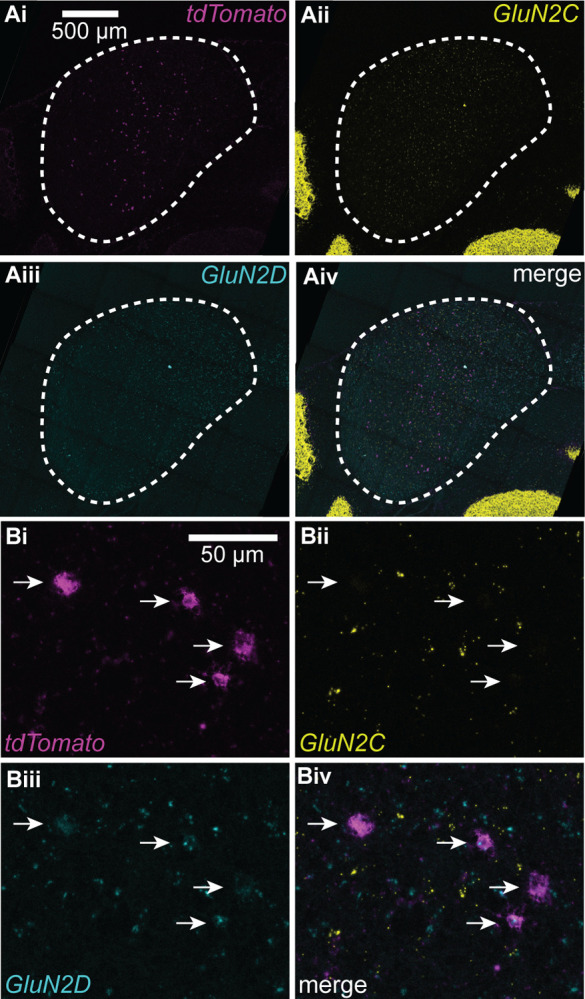
VIP neurons express GluN2D but rarely GluN2C mRNA **A**, 40x confocal images of IC brain slices showing expression of *tdTomato* mRNA **(i)**, which is expressed by VIP neurons in VIP-IRES-Cre × Ai14 mice, *GluN2C* mRNA **(ii)**, and *GluN2D* mRNA **(iii)**. **B**, 63x confocal images with white arrows indicating tdToma-to-positive neurons **(i)**. 8.1% of tdTomato positive neurons expressed *GluN2C* mRNA **(ii)** and 91.4% expressed *GluN2D* mRNA **(iii)**. *GluN2C* and *GluN2D* mRNA was also commonly observed in tdTomato-negative neurons, suggesting that these NMDAR subunits are expressed in multiple IC neuron types.

**Figure 4.**
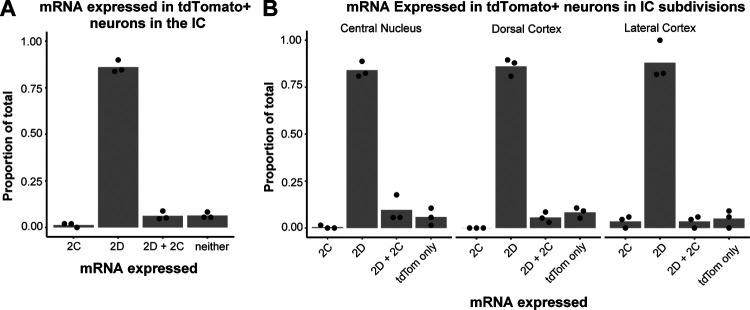
91.4% of VIP neurons express GluN2D mRNA. **A**, Quantification of mRNA expressed in tdTomato-positive (VIP) neurons in the IC. 1.0% of VIP neurons expressed only *GluN2C* mRNA, while 84.3% of VIP neurons expressed only *GluN2D* mRNA. 7.1% of VIP neurons expressed both *GluN2C* and *GluN2D* mRNA. 7.7% of VIP neurons did not express *GluN2C* or *GluN2D* mRNA. Individual data points represent the mean for individual mice. **B**, The central nucleus, lateral cortex, and dorsal cortex of the IC did not differ in their expression profiles of neurons expressing only *GluN2C* (0.8%, 0.0%, 3.8%, respectively), only *GluN2D* (83.7%, 85.0%, 86.5%), both *GluN2C* and *GluN2D* (7.9%, 6.2%, 3.8%), and neither *GluN2C*/*GluN2D* (7.6%, 8.8%, 5.8%). Individual data points represent the mean for individual mice.

Based on this, we hypothesized that IC VIP neurons in adult animals express GluN2C subunits. To test this hypothesis, we used the RNAScope assay to perform fluorescence in situ hybridization on IC brain slices from three VIP-IRES-Cre × Ai14 mice aged P45–47 (Figure 3A,B). Using probes for *tdTomato*, *GluN2C*, and *GluN2D* mRNA, we were surprised to find that 91.4% of VIP neurons expressed *GluN2D* and only 8.1% expressed *GluN2C*. More specifically, 84.3% of VIP neurons expressed only *GluN2D*, 1.0% of VIP neurons expressed only *GluN2C*, and 7.1% of VIP neurons expressed both. The remaining 7.7% of VIP neurons did not express *GluN2C* or *GluN2D* (Table 1, Figure 4A).

We also investigated whether NMDAR subunit expression differed among VIP neurons present in the three major IC subdivisions: central nucleus, dorsal cortex, and lateral cortex. We found that the percentage of VIP neurons expressing various NMDAR subunits was similar across all IC subdivisions (Table 2, Figure 4B). These results suggest that VIP neurons preferentially express *GluN2D* rather than *GluN2C* subunits, regardless of their location in the IC.

Importantly, our results also show that *GluN2C* and *GluN2D* mRNA is widely expressed in the IC (Figure 3A). This suggests that GluN2C and GluN2D-containing NMDARs are a prominent feature in the IC and likely have a significant and widespread impact on synaptic computations.

### VIP neurons also express GluN2A/B-containing NMDARs

**Figure 5.**
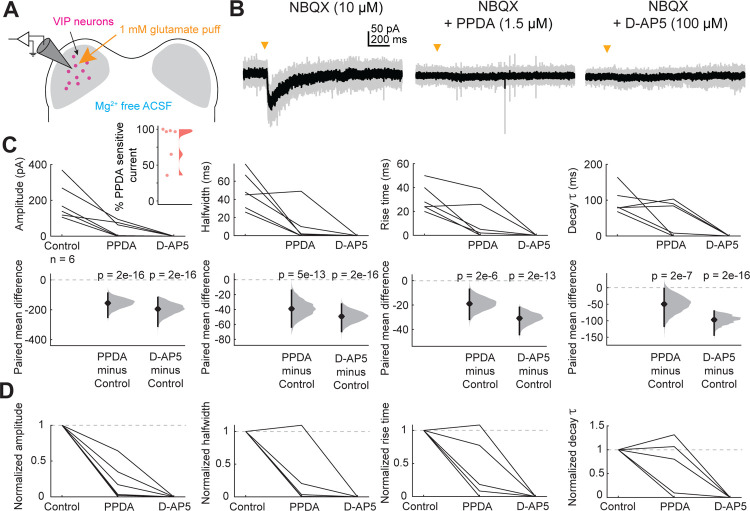
VIP neurons express GluN2A/B-containing NMDARs in addition to GluN2C/D-containing NMDARs **A**, Puffs of glutamate onto VIP neurons activated NMDARs. Pharmacology was used to examine the proportion of GluN2A/B and GluN2C/D containing receptors present on VIP neurons. **B**, An EPSC elicited by a brief (10 ms) puff of glutamate onto a VIP neuron was abolished by application of the GluN2C/D antagonist PPDA. **C**, PPDA decreased the amplitude (β = −155.49, 95% CI [−183.72, −127.25], *p* = 2e-16, n = 6), halfwidth (β = −39.03, 95% CI [−47.93, −30.13], *p* = 4.74e-13, n = 6), rise time (β = −18.61, 95% CI [−25.64, −11.57], *p* = 1.58e-06, n = 6), and decay τ (β = −54.23, 95% CI [−72.90, −35.58], *p* = 2.11e-07, n = 6) of the response to the glutamate puff. D-AP5 also decreased the amplitude (β = −194.83, 95% CI [−223.07, −166.60], *p* = 2e-16, n = 6), halfwidth (β = −49.282, 95% CI [−58.18, −40.38], *p* = 2e-16, n = 6), rise time (β = −30.87, 95% CI [−37.77, −23.97], *p* = 2.50e-13, n = 6), decay τ (β = −98.86, 95% CI [−117.15, −80.48], *p* = 2e-16, n = 6) of the response to the glutamate puff. D-AP5 completed abolished the response in all cells. **D**, Normalized amplitude, halfwidth, rise time, and decay τ plots from C.

We next investigated whether IC VIP neurons also express GluN2A or GluN2B-containing receptors. In the adult brain, GluN2A-containing NMDARs are more commonly found at synapses and confer more typical properties of the NMDA receptor, including sensitivity to Mg^2+^ block at rest (Siegler Retchless et al., 2012). To determine whether VIP neurons express GluN2A/B-containing NMDARs in addition to GluN2C/D-containing NMDARs, we targeted VIP neurons for whole-cell recordings and puffed glutamate in the presence of AMPAR antagonist NBQX (10 μM, Figure 5A). To examine the contributions of all NMDARs to the resulting current, we removed Mg^2+^ from the ACSF and replaced it with equimolar Ca^2+^ to maintain the overall concentration of divalent ions. We found that puffs of glutamate elicited much larger currents in the Mg^2+^-free condition than we observed in the earlier puff experiments (compare Figures 2C, 5C).

Next, we bath applied 1.5 μM PPDA, a GluN2C/D-specific antagonist, and as expected, we found that this significantly decreased the amplitude (LMM: β = −155.49, *p* = 2e-16), halfwidth (β = −39.03, *p* = 4.74e-13), rise time (β = −18.61, *p* = 1.58e-06), and decay time constant (β = −54.23, *p* = 2.11e-07) of the response, again suggesting that a portion of the NMDAR current in VIP neurons is mediated by GluN2C/D-containing NMDARs (Figure 5C). Next, we bath applied 100 μM D-AP5, an NMDAR antagonist that blocks NMDARs regardless of GluN2 subunit identity, and found that this completely abolished the response in all neurons (amplitude, β = −194.834, *p* = 2e-16). Overall, 5 out of 6 VIP neurons had both PPDA sensitive and insensitive currents, indicating activation of both GluN2A/B and GluN2C/D currents. On average in these cells, 82.0% ± 26.2% of the current was sensitive to PPDA (mean ± standard deviation, Figure 5 inset). One neuron had current that was completely abolished by PPDA indicating exclusive activation of GluN2C/D-containing receptors. These results suggest that individual VIP neurons can express multiple varieties of NMDARs that differ by subunit composition and are thus specialized for different roles in the cell.

### Commissural inputs to VIP neurons activate GluN2C/D-containing NMDA receptors

**Figure 6.**
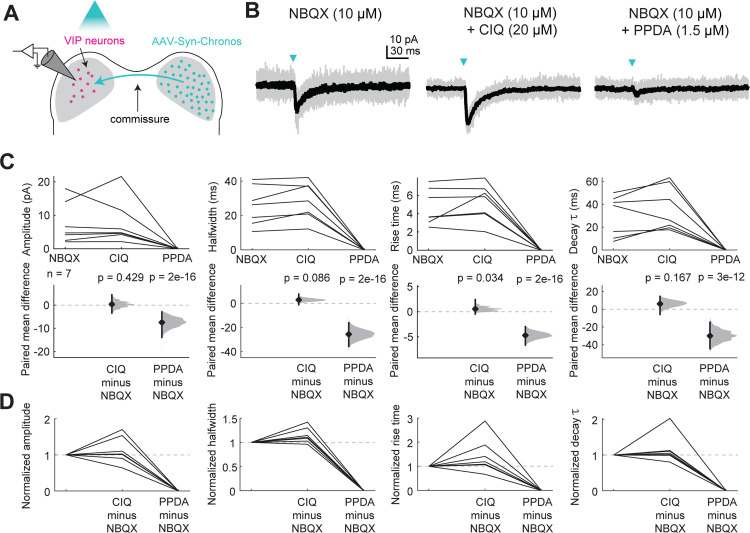
Commissural inputs to VIP neurons activate GluN2C/D-containing NMDARs at resting potential **A**, Viral injections in the contralateral IC drove Chronos expression in commissural projections to the IC. Optogenetic circuit mapping and pharmacology were used to examine postsynaptic responses in VIP neurons. **B**, An EPSC elicited by a brief (10 ms) flash of light (left) was enhanced by the GluN2C/D-specific positive allosteric modulator CIQ (middle) and diminished by the GluN2C/D-specific antagonist PPDA (right). **C**, CIQ did not alter the amplitude (LMM: β = −0.44, 95% CI [−1.53, 0.65], *p* = 0.43, n = 7), halfwidth (β = 2.47, 95% CI [−0.33, 5.27], *p* = 0.086, n = 7), or decay τ (β = 5.84, 95% CI [−2.40, 14.12], *p* = 0.17, n = 7) of the response, but did enhance the rise time (β = 0.51, 95% CI [0.042, 0.98], *p* = 0.034, n = 7). PPDA decreased the amplitude (β = 6.90, 95% CI [5.81, 7.99], *p* = 2e-16, n = 7), halfwidth (β = −26.89, 95% CI [−29.66, −24.11], *p* = 2e-16, n = 7), rise time (β = −4.92, 95% CI [−5.38, −4.45], *p* = 2e-16, n = 7), and decay τ (β = −31.02, 95% CI [−39.24, −22.76], *p* = 3.48e-12, n = 7) of the response. PPDA completely abolished the EPSPs, reducing all EPSP parameters to zero. **D**, Normalized amplitude, halfwidth, rise time, and decay τ plots from C.

We previously found that inputs from the contralateral IC via the IC commissure activate NMDARs on VIP neurons at resting potential (Goyer et al., 2019). To test whether these responses were also mediated by GluN2C/D-containing NMDAR receptors, we repeated these experiments in the presence of GluN2C/D-specific drugs. Commissural projections were labeled with the excitatory opsin Chronos by injecting AAV-Synapsin-Chronos-GFP (serotype 1, 5, or 9) into the right IC of VIP-IRES-Cre × Ai14 mice. 1.5 to 4 weeks later, we targeted whole-cell recordings to fluorescently labeled VIP neurons in the IC contralateral to the injection site. We found that even in the presence of AMPAR antagonist NBQX (10 μM), flashes of blue light elicited small EPSCs (−2 to −18 pA, Figure 6B). The GluN2C/D-specific positive allosteric modulator CIQ significantly increased the rise time (LMM: β = 0.51, *p* = 0.034) of the EPSC (Figure 6C), and the GluN2C/D-specific antagonist PPDA significantly decreased the amplitude (β = 6.90, *p* = 2e-16), halfwidth (β = −26.89, *p* = 2e-16), rise time (β = −4.92, *p* = 2e-16), and decay time constant (β = −31.02, *p* = 3.48e-12). These results support the hypothesis that commissural inputs to VIP neurons activate NMDARs containing GluN2C/D subunits.

### Activation of GluN2C/D-containing NMDARs facilitates temporal summation

**Figure 7.**
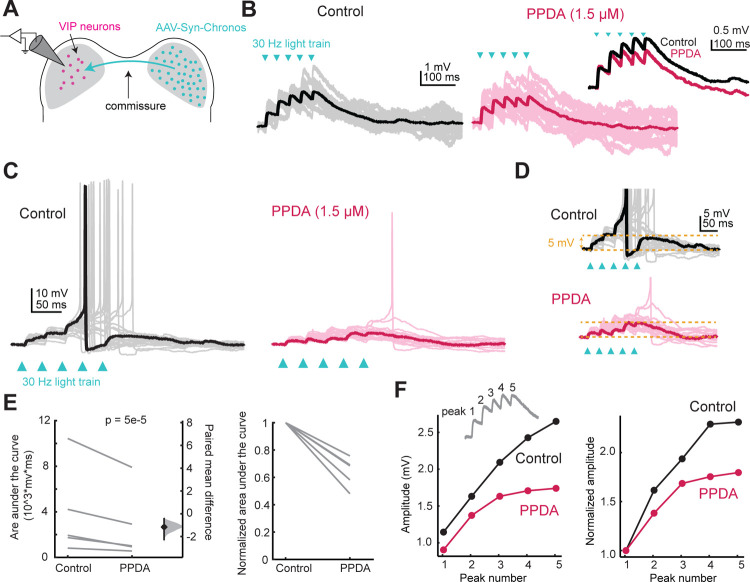
GluN2C/D-containing NMDARs facilitate temporal summation in VIP neurons **A**, Viral injections in the right IC drove Chronos expression in commissural projections to the left IC. Trains of optogenetic stimuli combined with pharmacology were used to examine the contributions of GluN2C/D-containing receptors to temporal summation. **B**, EPSP summation elicited by a 30 Hz train of light pulses (left) was decreased by application of the GluN2C/D-specific antagonist PPDA (right, comparison inset). **C**, Example of a cell in which summation of optogenetically elicited EPSPs reached action potential threshold, eliciting trains of action potentials. PPDA eliminated spiking in all but one sweep in this cell. **D**, Example traces from C scaled to demonstrate the differences in temporal summation between control and PPDA conditions. **E**, PPDA significantly decreased the area under the curve for the train of EPSPs (LMM: β = −70.370, 95% CI [−103.51, −36.86], *p* = 5.38e-05, n = 5), which can be seen in both the non-normalized (left) and normalized (right) plots. **F**, The peak amplitude of each EPSP was calculated from the baseline (see inset). The amplitude of the response increased with increasing peak number (LMM: β = 0.60, 95% CI [0.45, 0.76], *p* = 3.12e-14, n = 5) and decreased in the PPDA condition compared to the control condition (β = −0.37, 95% CI [−0.60, −0.13], *p* = 0.0018, n = 5), although the interaction between peak and condition was not statistically significant (β = 0.020, 95% CI [−0.19, 0.23], *p* = 0.85, n = 5).

Since NMDARs with GluN2C/D subunits have slower kinetics and conduct more current at resting membrane potential than NMDARs with GluN2A/B subunits (Paoletti et al., 2013), expression of GluN2C/D-containing NMDARs could have a particularly strong effect on the duration of excitation elicited by glutamatergic transmission. We therefore hypothesized that GluN2C/D-containing NMDARs in the IC facilitate temporal integration by widening the time window for integration of synaptic inputs. To test this hypothesis, we used optogenetics to stimulate commissural inputs to VIP neurons (Figure 7A). Trains of five light pulses at 30 Hz elicited temporal summation in all the cells tested, with the second through fifth EPSPs starting at a more depolarized potential than the first EPSP (Figure 7B). To test whether GluN2C/D-containing receptors facilitate this temporal summation, we next elicited trains of input in the presence of the GluN2C/D-containing NMDAR antagonist PPDA (1.5 μM). The amount of temporal summation in each cell was assessed first by calculating the area under the curve for the EPSP train, and PPDA significantly decreased the area under the curve (LMM: β = −70.37, *p* = 5.38e-05) (Figure 7E). In one cell, the light train elicited temporal summation great enough to elicit an action potential in the control condition, and this was impaired in the PPDA condition, where the cell only reached action potential threshold in one trial (Figure 7C,D). We also assessed temporal summation by comparing the peak amplitudes of each of the five EPSPs elicited by the train stimuli in the control and PPDA conditions. The amplitude of the peaks significantly increased during the train (LMM: β = 0.60, 95% CI [0.45, 0.76], *p* = 3.12e-14, n = 5) and significantly decreased following application of PPDA (β = −0.37, 95% CI [−0.60, −0.14], *p* = 0.0018, n = 5) (Figure 7F). These results show that GluN2C/D-containing NMDARs strongly enhance the time window for synaptic integration in VIP neurons. Since our in situ hybridization data showed that GluN2C/D subunits are common in VIP neurons and many other neurons throughout the IC (Figures 3,4), the enhancement of temporal summation by GluN2C/D-containing NMDARs throughout the IC could play an important role in supporting the transition from temporal coding to rate coding for auditory stimuli.

## Discussion

In this study, we found that IC VIP neurons express GluN2D-containing NMDARs that significantly contribute to postsynaptic responses at resting membrane potentials. By combining optogenetics with whole-cell recordings, we showed that NMDARs in VIP neurons are activated at resting potential by commissural projections and by ascending inputs from the AVCN. By using puffs of glutamate and whole-cell recordings combined with GluN2C/D-specific pharmacology, we showed that GluN2C/D-containing NMDARs activate at resting potential in VIP neurons. We demonstrated that 91% of VIP neurons express mRNA for the GluN2D subunit, which confers less sensitivity to Mg^2+^ block and slower kinetics than NMDARs with GluN2A/B subunits. We also found that *GluN2C* and *GluN2D* mRNA is prevalent throughout the IC, including in many non-VIP neurons, suggesting that GluN2C/D-containing NMDARs exert widespread influence over excitatory postsynaptic responses in the IC. Additionally, we demonstrated that VIP neurons can also express GluN2A/B-containing receptors, suggesting that different types of NMDARs may play distinct roles in these cells. Finally, we showed that GluN2C/D-containing receptors facilitate temporal summation in VIP neurons by lengthening the window for synaptic integration. Thus, NMDAR diversity shapes how ascending and commissural circuits drive synaptic integration in the IC.

### VIP neurons express NMDARs with GluN2D subunits

One of the most intriguing findings from our study was that VIP neurons predominately express GluN2D-containing NMDARs rather than GluN2C. This result was unexpected for two reasons: first, in most brain regions, expression of GluN2D is developmentally regulated and disappears in adulthood (Monyer et al., 1994; Wenzel et al., 1996). Second, adult expression of GluN2D is conventionally thought to be restricted to extrasynaptic locations, for example, in dorsal horn neurons (Momiyama, 2000) and Golgi cells of the cerebellum (Misra et al., 2000; Brickley et al., 2003). However, NMDARs are highly motile and can rapidly diffuse between extrasynaptic and synaptic sites (Tovar and Westbrook, 2002), as occurs during learning. During induction of NMDAR-LTP in the medial perforant path of the dentate gyrus, GluN2D-containing NMDARs are trafficked to the synapse where they contribute to synaptic transmission (Harney et al., 2008). Based on this, we hypothesize that GluN2D-containing receptors rapidly diffuse to synaptic locations on VIP neurons during learning. An alternative explanation is that GluN2D-containing NMDARs are natively found in the synapse and do not undergo a GluN2D-to-GluN2A/C switch during development.

Additionally, while we show here that many NMDARs on VIP neurons contain the GluN2D subunit, the full subunit composition of the receptor remains unknown. NMDARs are tetrameric receptors made up of four distinct subunits, including two obligatory GluN1 subunits and a combination of GluN2A-D and/or GluN3 subunits (Traynelis et al., 2010). Our in situ hybridization results showed that 91% of VIP neurons express GluN2D mRNA. This could give rise to NMDARs that are diheteromeric with two GluN1 subunits and two GluN2D subunits. However, GluN1-GluN2D receptor assemblies have much slower kinetics than we observed in our study (decay τ = 10–100 ms), with decay time constants on the order of 2 seconds (Vicini et al., 1998). An alternative hypothesis is that GluN2D-containing receptors in VIP neurons are triheteromeric GluN1-GluN2B-GluN2D complexes. These receptors display properties that are intermediate to those of GluN1-GluN2B and GluN1-GluN2D receptors, including slightly faster kinetics than GluN1-GluN2D assemblies (Yi et al., 2019) and a reduced sensitivity to Mg^2+^ block compared to GluN1-GluN2B assemblies (Huang and Gibb, 2014). In addition, receptors containing GluN2B tend to be more motile than NMDARS with GluN2A subunits (Groc et al., 2006). Thus, this proposed GluN1-GluN2B-GluN2D assembly may also help explain how GluN2D-containing receptors contribute to synaptic transmission in mature VIP neurons.

### Distribution of NMDARs on VIP neurons is pathway-specific

Previous work from our lab showed that NMDAR activation at resting potential on VIP neurons occurs with commissural inputs but not inputs from the DCN (Goyer et al., 2019). The results presented here show that AVCN projections to VIP neurons also activate NMDARs at resting potential. This suggests that the distribution of NMDARs on VIP neuron synapses is pathway-dependent, with commissural and AVCN inputs targeting synapses that are enriched in GluN2D-containing receptors while DCN inputs target synapses predominated by AMPARs and possibly GluN2A/B-containing NMDARs. Such differences in receptor distribution could underlie functional differences between these synapses, as has been seen in other brain regions such as neocortex (Kumar and Huguenard, 2003). For example, commissural and/or AVCN inputs may be integrated over longer time scales than those from the DCN, and a neuron may require more DCN inputs within a smaller time window to generate a postsynaptic spike.

NMDAR subtype distribution might also affect synaptic plasticity. GluN2A/B-containing NMDARs have been well-studied for their role in the initiation of long-term potentiation (LTP) (Paoletti et al., 2013). In the IC, previous studies show that electrical stimulation of the lateral lemniscus can induce LTP in IC neurons (Hosomi et al., 1995), and NMDARs are required for this process (Zhang and Wu, 2000; Wu et al., 2002). Our glutamate puff experiments showed that VIP neurons also express GluN2A/B-containing NMDARs, indicating that at least some VIP neuron synapses may exhibit AMPAR-dependent LTP (Malenka and Nicoll, 1993; Rebola et al., 2010; Hunt and Castillo, 2012). It will be important for future studies to determine the contributions of GluN2D trafficking and GluN2A/B expression to synaptic plasticity in the IC.

### Commissural inputs to VIP neurons activate GluN2D-containing receptors

We showed that GluN2D-containing NMDARs are activated on VIP neurons by commissural inputs. Commissural inputs primarily serve to modulate IC neuron responses. For example, deactivation of the contralateral IC via cooling results in heterogeneous changes in firing rate and local field potentials in the ipsilateral IC (Orton et al., 2012). Similarly, injection of kainic acid into the contralateral IC to block commissural projections results in heterogeneous changes in neuron responses to monaural and binaural sounds (Malmierca et al., 2005) and to neuron frequency response areas in the ipsilateral IC (Malmierca et al., 2003). While the circuit mechanisms that underlie the modulatory role of commissural fibers are not well understood, GluN2D-containing receptors may play a role in this process by enhancing the time window over which commissural and other ascending/descending inputs are integrated, which could enhance the ability of commissural inputs to influence IC neuron responses to auditory stimuli. In addition, commissural inputs in the IC are important for sharpening azimuthal sound localization (Orton et al., 2016). By enhancing the time window for synaptic integration of commissural inputs, GluN2D-containing receptors may enhance the influence of the ipsilateral ear on sound localization computations in the IC.

### GluN2D-containing receptors may facilitate the transition from temporal to rate coding in the IC

Here, we show that GluN2D-containing NMDARs facilitate temporal integration in the IC and are activated by ascending inputs. We therefore propose that GluN2C/D-containing NMDARs may be important for the shift from temporal to rate coding of amplitude modulated (AM) stimuli in the IC. Auditory structures primarily encode changes in amplitude envelopes in two ways: using temporal codes, where neurons phase-lock their firing to the modulation waveform, and using rate codes, where a neuron’s firing rate changes based on the frequency of the amplitude modulation (AM). While early auditory structures such as the cochlear nucleus primarily use temporal codes for AM stimuli (Rhode and Greenberg, 1994), neurons in auditory cortex primarily use rate codes (Yin et al., 2011). Since many IC neurons only phase lock to lower AM modulation frequencies (Rees and Møller, 1983; Rees and Palmer, 1989; Krishna and Semple, 2000) but exhibit strong and diverse dependencies of firing rate on changes in AM modulation frequency (Krishna and Semple, 2000; Nelson and Carney, 2007; Geis and Borst, 2009; Kim et al., 2020), the IC has been proposed as a critical site for the temporal-to-rate code transition that occurs between brainstem and cortex.

Our data suggest that activation of GluN2C/D-containing NMDARs enhances temporal summation of synaptic inputs, which could turn phase-locked ascending inputs from the lower brainstem into the action potentials that form a rate code. In support of this hypothesis, Zhang and Kelly (2001) found that blocking NMDARs reliably flattened rate coding in IC neurons while leaving temporal coding of AM stimuli intact (Zhang and Kelly, 2001). However, the effects of specific subunit-containing NMDARs on this process have not been studied. In future studies, we will use pharmacology and targeted in vivo recordings to test how GluN2C/D-containing receptors shape the temporal-to-rate coding transition in the IC.

## Materials and Methods

### Animals

All experiments were approved by the University of Michigan Institutional Animal Care and Use Committee and were in accordance with NIH guidelines for the care and use of laboratory animals. Animals were kept on a 12-hour day/night cycle with ad libitum access to food and water. To visualize VIP neurons, VIP-IRES-Cre mice (VipTM1(cre)Zjh/J, Jackson Laboratory, stock #010908) (Taniguchi et al., 2011) were crossed with Ai14 reporter mice (B6.Cg-Gt(ROSA)26SorTM 14(CAG–tdTomato)Hze/J, Jackson Laboratory, stock #007914) (Madisen et al., 2010) so that the fluorescent protein tdTomato was expressed only in VIP neurons (Beebe et al., 2022). Since mice on the C57BL/6J background undergo age-related hearing loss after 3 months of age (Zheng et al., 1999), all experiments were performed on mice between postnatal days (P)30 – 66.

### Brain slice preparation

Whole-cell patch-clamp recording were performed in acutely prepared IC slices from VIP-IRES-Cre × Ai14 mice. Male (n = 18) and female (n = 15) mice aged P30 – P66 were used. Mice were deeply anesthetized with isoflurane and then rapidly decapitated. The brain was removed and the IC was dissected in 34 °C artificial cerebrospinal (ACSF) solution containing (in mM): 125 NaCl, 12.5 glucose, 25 NaHCO_3_, 3 KCl, 1.25 NaH_2_PO_4_, 1.5 CaCl_2_, 1 MgSO_4_, 3 sodium pyruvate and 0.40 L-ascorbic acid (Acros Organics), bubbled with 5% CO2 in 95% O2. 200 μm coronal slices containing the IC were cut using a vibrating microtome (VT1200S, Leica Biosystems). Slices were incubated at 34 °C for 30 min in ACSF bubbled with 5% CO2 in 95% O2 and then placed at room temperature for at least 30 min before initiating recordings. Recordings were targeted to tdToma-to-expressing VIP neurons in the central nucleus of the IC using a Nikon FN1 or Olympus BX51 microscope. All chemicals were obtained from Thermo Fisher Scientific unless otherwise noted.

### Voltage-clamp electrophysiological recordings

Slices were placed in a recording chamber and continuously perfused at a rate of ~2 ml/min with 34 °C ACSF bubbled in 5% CO2/95% O2. Whole-cell voltage-clamp recordings were performed with an Axopatch 200A patch clamp amplifier (Axon Instruments). For each recording, series resistance compensation was performed using 80% prediction and 80% correction, and whole cell capacitance was compensated. Series resistance for all neurons included in this study was <15 MΩ. Data were low-pass filtered at 10 kHz, sampled at 50 kHz with a National Instruments PCIe-6343 data acquisition board, and acquired using custom software written in IgorPro. Recording pipettes were pulled from borosilicate glass capillaries (outer diameter 1.5 mm, inner diameter 0.86 mm, Sutter Instrument) with a P-1000 microelectrode puller (Sutter Instrument) and filled with an internal solution containing (in mM): 115 CsOH, 115 D-gluconic acid, 7.76 CsCl, 0.5 EGTA, 10 HEPES, 10 Na2 phosphocreatine, 4 MgATP, 0.3 NaGTP, supplemented with 0.1% biocytin (w/v), pH adjusted to 7.4 with CsOH and osmolality to 290 mmol/kg with sucrose. Voltage clamp recordings were not corrected for the liquid junction potential. All electrophysiological recordings were targeted to neurons in the central nucleus of the IC, but it is possible a small number of recordings were performed in the dorsal cortex of the IC.

To apply glutamate puffs to brain slices, we pulled puffer pipettes from borosilicate glass (outer diameter 1.5 mm, inner diameter 0.86 mm, Sutter Instrument) to a resistance of 3.5 – 5.0 MΩ using a P-1000 microelectrode puller and filled them with 300 μM glutamate (Sigma, #G5889) dissolved in a vehicle solution containing (in mM): 125 NaCl, 3 KCl, 12.5 glucose and 3 HEPES. The solution was balanced to a pH of 7.40 with NaOH. Puffer pipettes were connected to a pressure ejection system built based on the OpenSpritzer design (Forman et al., 2017). The tips of puffer pipettes were placed near the soma of the recorded cell, and 10 ms puffs were presented either 10 ms or 30 ms apart (30 ms for drug conditions) with 5 puffs presented per condition. Puffs containing only the vehicle solution did not elicit any response in the neurons tested (Figure 2E–H).

To examine whether NMDAR activation in VIP neurons at resting membrane potential is mediated by NMDARs containing GluN2C/D subunits, we performed whole-cell patch-clamp recordings targeted to VIP neurons as described above. To isolate the contribution of NMDARs, recordings were performed in the presence of 10 μM NBQX, an AMPAR antagonist. Glutamate was puffed onto VIP neurons during bath application of 1.5 μM PPDA (Hello Bio, #HB0531), a GluN2C/D specific antagonist (Lozovaya et al., 2004; Li et al., 2020; Jing et al., 2022), or 20 μM CIQ (Hello Bio, HB0197), a GluN2C/D specific positive allosteric modulator (Feng et al., 2014; Zhang et al., 2014; Nouhi et al., 2018; Liu et al., 2021). The concentrations of PPDA and CIQ used throughout this study are based on previous studies that used PPDA (Lozovaya et al., 2004; Li et al., 2020; Jing et al., 2022) or CIQ (Feng et al., 2014, 2014; Nouhi et al., 2018; Liu et al., 2021). All drugs were washed in for 10 minutes before testing how the drugs affected responses to glutamate puffs.

CIQ and PPDA were prepared in 0.04% and 0.003% DMSO (volume/volume), respectively. To test for vehicle effects, we performed control experiments where puff-elicited EPSCs were compared between control and DMSO (vehicle) conditions. No differences in puff-elicited EPSCs were found after DMSO wash-in (Figure 2E–F).

### Mg^2+^-free voltage clamp recordings

To determine what proportion of NMDARs on VIP neurons contain GluN2C/D subunits vs GluN2A/B subunits, we performed whole-cell patch-clamp recordings targeted to VIP neurons as described above, except immediately prior to patching brain slices were transferred to a Mg^2+^-free ACSF solution containing (in mM): 125 NaCl, 12.5 glucose, 25 NaHCO_3_, 3 KCl, 1.25 NaH_2_PO_4_, 2.5 CaCl_2_, 0 MgSO_4_, 3 sodium pyruvate and 0.40 L-ascorbic acid (Acros Organics), bubbled with 5% CO2 in 95% O2. This solution removed Mg^2+^ block from NMDA receptors. To isolate the contribution of NMDARs, recordings were performed in the presence of 10 μM NBQX. To determine whether GluN2A/B-containing NMDARs were present on VIP neurons, we first performed control glutamate puffs and then puffs after bath application of 1.5 μM PPDA, followed by puffs after bath application of 100 μM D-AP5, a broad NMDAR antagonist. All drugs were washed in for 10 minutes before testing how the drugs affected the response to glutamate puffs.

### Intracranial virus injections

Mice used for intracranial injections of recombinant adeno-associated viruses (AAVs) were between ages P26 – 39. Mice were anesthetized with 1–3% isoflurane (Piramal Critical Care, # NDC 66794-017-25) and body temperature was maintained using a homeothermic heating pad. To minimize postoperative pain, an injection of carprofen (5 mg/kg, CarproJect, Henry Schein Animal Health) was administered subcutaneously. The scalp was shaved using scissors and an incision was made along the midline of the scalp to expose the skull. The injection site was identified using previously validated coordinates for either the IC (all coordinates are relative to lambda; in μm: X = −900, Y = 1000, X = 2250–1500 at 250 intervals, and X = −900, Y = 1250, X = 2250–1750 at 250 intervals) or the AVCN (in μm: X: −485, Y = 2455, Z = 2950 – 4750 at 200 intervals) and a craniotomy was drilled using a micromotor drill (K.1050, Foredom Electric Co.) with a 0.5 mm burr (Fine Science Tools). Glass injection pipettes were pulled from capillary glass (Drummond Scientific Company) using a P-1000 microelectrode puller (Sutter Instruments) and cut on a diagonal for a beveled opening of ~20 μm. Pipettes were first backloaded with mineral oil and then front filled with AAV1.Syn.Chronos-GFP.WPRE.bGH (Addgene, #59170-AAV1, titer: 1.4e13, 2.2e13), AAV5.Syn.Chronos-GFP.WPRE. bGH (Addgene, #59170-AAV5, titer: 5.3e12), or AAV9.Syn.Chronos-GFP-WPRE.bGH (University of North Carolina Vector Core, Addgene plasmid #59170, titer: 4.5e12) (Klapoetke et al., 2014). The virus was injected using a NanoJect III nanoliter injector (Drummond Scientific Company) connected to an MP-285 micromanipulator (Sutter Instruments). IC injections were made in two penetrations along the medial-lateral axis that were 250 μm apart, and viral deposits were made at 250 μm intervals along the ventral-dorsal axis for a total of 4 deposits at the more medial site and 3 deposits at the more lateral site. 20 nL of virus was deposited per injection, for a total of 150 nL virus injected per IC. VCN injections were made in two penetrations along the medial-lateral axis that were 200 μm apart, for a total of 2 20nL deposits and a total of 40 nL virus injected per VCN. After the injections were completed, the scalp was closed either by suturing with Ethilon 6–0 (0.7 metric) nylon sutures (Ethicon USA, LLC) or applying Vetbond (3M, #1469SB) on top of the closed incision. For postoperative analgesia, 0.5 mL of 2% Lidocaine hydrochloride jelly (Akorn Inc) was placed on top of the sutures. Mice were observed for 1 hr for indications of pain or distress and then returned to the vivarium once they were ambulatory. Mice were monitored daily until sutures fell out and the wound was completely healed. Sutures remaining on the 10th post-operative day were manually removed.

### Current-clamp electrophysiological recordings

Slices were placed in a recording chamber and continuously perfused at a rate of ~2 ml/min with 34 °C oxygenated ACSF. Whole-cell current-clamp recordings were performed with a BVC-700A patch clamp amplifier (Dagan Corporation). Data were low-pass filtered at 10 kHz, sampled at 50 kHz with a National Instruments PCIe-6343 data acquisition board, and acquired using custom software written in IgorPro. Recording pipettes were pulled from borosilicate glass pipettes (outer diameter 1.5 mm, inner diameter 0.86 mm, Sutter Instrument) with a P-1000 microelectrode puller (Sutter Instrument) and filled with an internal solution containing (in mM): 115 K-gluconate, 7.73 KCl, 0.5 EGTA, 10 HEPES, 10 Na_2_phosphocreatine, 4 MgATP, 0.3 NaGTP, supplemented with 0.1% biocytin (w/v), pH adjusted to 7.3 with KOH and osmolality to 290 mmol/kg with sucrose. All membrane potentials for current-clamp recordings were corrected for an 11 mV liquid junction potential.

### Optogenetics

Current-clamp recordings were conducted 2 – 4 weeks after virus injections to allow time for Chronos expression. Recordings were conducted as described above, except that brain slices were prepared and incubated under red light to limit Chronos activation. To verify the virus injection location in IC injections, fluorescence was visualized under the microscope during the recording session. In most cases, AVCN injections were verified during post hoc imaging of fixed AVCN sections. Recordings were targeted to neurons contralateral to the injection site.

Chronos was activated using 2–10 ms pulses of 470 nm light emitted by a blue LED coupled to the epi-fluorescence port of the microscope and delivered to the slice through a 0.80 NA 40x water immersion objective. Blue light flashes illuminated the entire field of the 0.80 NA 40x objective, corresponding to optical power densities of 6 to 48 mW/mm^2^. Optical power was set using the minimum stimulation needed to elicit an EPSP from the recorded neuron. Recording sweeps with light flashes were repeated 10–30 times with 10 ms between light flashes.

### RNAscope In situ hybridization

To quantify the presence of *GluN2C* and *GluN2D* mRNA in VIP neurons, we performed fluorescent in situ hybridization for *tdTomato*, *GluN2C*, and *GluN2D* using the RNAscope fluorescent multiplex detection kit (Advanced Cell Diagnostics, catalog # 320850). Our methods were identical to those previously described (Beebe et al., 2022) and followed manufacturer recommendations (Wang et al., 2012). Briefly, one male (P47) and two female (P45) mice were deeply anesthetized with isoflurane and brains were rapidly removed and frozen on dry ice. Brains were placed in a −80 °C freezer until the day of slicing. Prior to slicing, brains were equilibrated at −20 °C for 1 hour. Brains were sliced into 15 μM sections using a cryostat at −20 °C and sections were mounted on Superfrost Plus slides (Fisher Scientific, catalog # 22037246). For each mouse, five representative sections were chosen spanning the caudal—rostral axis. Sections were fixed using 10% neutral buffered formalin (Sigma-Aldrich, catalog #HT501128) and dehydrated using repeated washes in 50%−100% ethanol. Slides were dried using a Kim wipe and a hydrophobic barrier was drawn around each section. Slices were next incubated in hydrogen peroxide for 10 minutes at room temperature, followed by application of Protease IV for 30 minutes.

Probes for *tdTomato*, *GluN2C*, and *GluN2D* (all experimental slices except controls), along with positive and negative controls (one control slice each), were applied to slices and incubated for 2 hours at 40 °C. The probes were amplified three times for 30 minutes each time at 40 °C using AMP 1–3. The signal was developed using the HRP for each channel and then opal dyes diluted at 1:1000 were assigned for each channel: *tdTomato* was assigned to Opal 690 (Akoya Bioscience, catalog #FP1497001KT), *GluN2C* was assigned to Opal 570 (Akoya Bioscience, catalog #FP1488001KT), and *GluN2D* was assigned to Opal 520 (Akoya Bioscience, catalog #FP-1487001KT). Slices were counterstained with DAPI and coverslipped with ProLong Gold antifade mountant (Fisher Scientific, catalog #P36934). Slices were imaged within 1 week of performing the assay using the 0.75 NA 20X objective on a Leica TCS SP8 laser scanning confocal microscope (Leica Microsystems) at 2 μm depth intervals. Emission wavelengths were adjusted for each channel as follows: DAPI (405 nm laser, 410–441 nm), *GluN2D* (488 nm laser, 491–528 nm), *GluN2C* (552 nm laser, 565–583 nm), *tdTomato* (638 nm laser, 690–727 nm).

Co-localization of *GluN2C* and *GluN2D* in *tdTomato*-positive neurons was quantified manually using Neurolucida 360 (MBF Bioscience). One side of the IC (left or right) was selected randomly for quantification in each slice. *tdTomato*-positive neurons were first identified by placing a marker on top of the cell, and then *GluN2C* and *GluN2D* fluorescence was quantified separately for each marked cell. Cells were considered positive for *GluN2C* or *GluN2D* when one or more puncta co-localized with the *tdTomato* fluorescence. All *tdTomato*-positive neurons counted co-labeled with DAPI.

Subdivisions of the IC were determined for each IC slice used in the above analysis by comparison to a reference series of sections from a control C57BL/6J mouse aged P49 that were immunolabeled for GAD67 and GlyT2. This pattern of labeling is routinely used to identify IC subdivisions (Choy Buentello et al., 2015; Beebe and Schofield, 2021), including in our previous studies (Silveira et al., 2020; Anair et al., 2022).

### Analysis of electrophysiological recordings

Amplitude, halfwidth, rise time, and decay time constant measurements were made using custom algorithms in Igor Pro 8 (Wavemetrics). Voltage-clamp data were low-pass filtered at 3 kHz prior to analysis, except for the commissural optogenetic experiment (Figure 6), where data were low-pass filtered at 1 kHz and the following function was fit to each response, which was then analyzed:

$$f\left(x\right)=y_{0}+\text{amp}\left(1-e^{\frac{-\left(x-x_{0}\right)}{\tau_{\text{rise}}}}\right)*e^{\frac{-\left(x-x_{0}\right)}{\tau_{\text{decay}}}}$$

For the temporal summation experiment (Figure 7), the peak of each EPSP in the train was calculated as the maximum value between light pulses. The plot in Figure 7F depicts the averages for cells in the control and PPDA conditions.

### Statistical analyses

All data were analyzed using the estimation statistics approach (Bernard, 2019; Calin-Jageman and Cumming, 2019) which highlights the importance of effect sizes and confidence intervals rather than p values. Our approach to this method has been detailed previously (Rivera-Perez et al., 2021). Data analysis was performed using custom algorithms and statistical packages in Igor Pro 8 (Wavemetrics), MATLAB R2021a (MathWorks), and R 4.1.0 (The R Project for Statistical Computing). The electrophysiological data shown here involved repeated measures from individual cells, and this data is shown using Gardner-Altman estimation plots (two groups) or Cumming estimation plots (three groups). These plots were made using custom MATLAB scripts but were modeled from the DABEST package (Ho et al., 2019). The parallel coordinate plots show the responses of individual cells for each condition, and these are accompanied by the paired mean difference between each drug condition and the control. Linear mixed model (LMM) analyses were conducted in R using the “lme4” and “lmerTest” packages (Bates et al., 2015; Kuznetsova et al., 2017).

## Figures and Tables

**Table 1.** The number and percentage of tdTomato positive cells that expressed GluN2C and GluN2D mRNA using an RNAScope in situ hybridization assay. Data is from a series of coronal IC sections collected from three VIP-IRES-Cre × Ai14 mice aged P45–47.

Animal	Slice (n)	No. of *tdTomato+*	No. of *GluN2D+*	No. of *GluN2C+*	% *tdTomato+ GluN2D+*	% *tdTomato+ GluN2C+*
**Mouse 1**	A (caudal)	42	36	3	85.7%	7.1%
	B (rostral)	3	3	0	100.0%	0.0%
	C (caudal)	52	49	4	94.2%	7.7%
	D (rostral)	4	4	0	100.0%	0.0%
	E (caudal)	48	47	2	97.9%	4.2%
	**Total**	**149**	**139**	**9**	**93.3%**	**6.0%**
**Mouse 2**	A (rostral)	3	3	0	100.0%	0.0%
	B (medial)	13	10	2	76.9%	15.4%
	C (medial)	26	26	4	100.0%	15.4%
	D (caudal)	71	68	9	95.8%	12.7%
	**Total**	**113**	**107**	**15**	**94.7%**	**13.3%**
**Mouse 3**	A (medial)	70	62	2	88.6%	2.9%
	B (caudal)	94	83	10	88.3%	10.6%
	C (rostral)	10	9	1	90.0%	10.0%
	D (caudal)	85	76	5	89.4%	5.9%
	**Total**	**259**	**230**	**18**	**88.8%**	**6.9%**
**Grand total**		**521**	**476**	**42**	**91.4%**	**8.1%**

**Table 2.** The number and percentage of tdTomato positive cells in the three major IC subdivisions (central nucleus, lateral cortex, and dorsal cortex) that expressed GluN2C and GluN2D mRNA using an RNAScope in situ hybridization assay. Data is from a series of coronal IC sections collected from three VIP-IRES-Cre × Ai14 mice aged P45–47.

Location	Mouse	No. of *tdTomato+*	No. of *GluN2D+*	No. of *GluN2C+*	% *tdTomato+ GluN2D+*	% *tdTomato+ GluN2C+*
**Central nucleus**	1	89	84	5	94.4%	5.6%
	2	68	67	12	98.5%	17.6%
	3	199	175	14	87.9%	7.0%
	**Total**	**356**	**326**	**31**	**91.6%**	**8.7%**
**Dorsal cortex**	1	47	42	4	89.4%	8.5%
	2	28	25	1	89.3%	3.6%
	3	38	36	2	94.7%	5.3%
	**Total**	**113**	**103**	**7**	**91.2%**	**6.2%**
**Lateral cortex**	1	13	13	0	100.0%	0.0%
	2	17	15	2	88.2%	11.8%
	3	22	19	2	86.4%	9.1%
	**Total**	**52**	**47**	**4**	**90.4%**	**7.7%**
**Grand total**		**521**	**476**	**42**	**91.4%**	**8.1%**
